# Evaluation of the masking ability, marginal adaptation, and fracture resistance of screw-retained lithium disilicate implant-supported crowns cemented to titanium bases versus preparable abutments

**DOI:** 10.1186/s12903-023-03281-8

**Published:** 2023-08-30

**Authors:** Ahmad Waled Mohamad Kordi, Abdallah Ibrahim Salman, Nayrouz Adel Metwally, Mohamed Moataz Khamis

**Affiliations:** 1 Clinical Master of Oral Implantology Program, Faculty of Dentistry, Alexandria University, Alexandria, Egypt; 2 Department of Prosthodontics, Faculty of Dentistry, Alexandria University, 9 Hussein Shereen Street, Louran, Alexandria Egypt

**Keywords:** Implant restoration, Screw cement retained, Masking ability, Marginal adaptation, Fracture resistance, Preparable abutment, Ti base

## Abstract

**Background:**

Straight preparable abutments and titanium bases (ti-base) can be used to support single-unit screw-retained lithium disilicate implant-supported restorations. The choice between using both abutments depends on many factors. The purpose of this in vitro study was to compare the masking ability, marginal adaptation, and fracture resistance of screw-retained lithium disilicate implant-supported crowns cemented to straight preparable abutments and ti-bases.

**Methods:**

Twenty laboratory implant analogs (Straumann Bone Level; Straumann AG) were randomly divided into 2 groups (n = 10 each) according to the type of the abutment used. Preparable abutment group and ti-base group. Lithium disilicate crowns were used to restore the specimens. All specimens were subjected to thermocycling (from 5 to 55 °C for 2000 cycles) followed by cyclic loading (120 000 cycles). The vertical marginal gap between the abutment finish line and the most apical part of the crown was measured in (µm) by using a stereomicroscope after cementation and after thermocycling and cyclic loading. A spectrophotometer was used to evaluate the masking ability of the specimens after cementation. The load required to fracture the crowns was measured in Newtons (N) by using a universal testing machine after thermocycling and cyclic loading. The Shapiro-Wilk test of normality was used. The appropriate statistical test was used.

**Results:**

Regarding the masking ability, the color difference (∆E) showed no statistically significant difference between the ti-base group (2.6 ± 0.2) and the preparable abutment group (2.6 ± 0.3) (*P* = .888). The average of the microgap values (µm) was greater in ti-basegroup after cementation (13.9 ± 9.2) than preparable group (7.63 ± 1.78) with no statistically significant difference between the 2 groups (*P* = .49). After cyclic loading and thermocycling, the average microgap values (µm) was significantly greater in the ti base group (21.3 ± 7.4) than in preparable group (13.3 ± 1.5) (*P* = .02). The load required to fracture the specimens was greater in the preparable group (1671.5 ± 143.8) than in the ti-base group (1550.2 ± 157.5) with no statistically significant difference between the 2 groups (*P* = .089).

**Conclusion:**

The abutments used in the present study did not compromise the masking ability of the screw-retained lithium disilicate implant supported crowns. Moreover, the crowns cemented to preparable abutments had better marginal adaptation and higher fracture resistance when compared to those cemented to ti-bases.

**Clinical implications:**

Straight preparable abutments are considered as an alternative to the ti-bases when restoring single screw-retained lithium disilicate implant-supported crowns with comparable fracture resistance, marginal adaptation, and masking ability.

## Background

Monolithic lithium disilicate cement-retained implant restorations having a screw access channel combine the advantages of both screw and cement retention [1]. They are usually cemented to titanium-bases (ti-base) when fabricated by computer-aided design and computer-aided manufacturing (CAD-CAM). However, straight preparable titanium stock abutments have been well documented as an alternative to ti-bases [2, 3]. Preparable abutments are stock abutments that can be prepared or modified inside the patient mouth or on the model. The advantages of using preparable abutments is that they have varying heights and diameters providing additional surface area to improve the crown/abutment bond strength. Minor adjustments and preparations can be made if needed. In case of unavailability of a scan body, abutments can be scanned directly and hence there is no need for a special implant library in the CAD software program. The limitations of using preparable abutments as an alternative to ti-bases include the need for intraoral crown cementation and the inaccessibility of the abutment shoulder scanning in case of subgingival finish lines [4, 5].

A specially designed ti-base and computer aided design (CAD) block are used with the CAD-CAM system where the block is provided with a prefabricated screw access channel having an anti-locking slot accurately fitting the ti-base [4]. This combination is intended to provide extraoral cementation with high precision. However, the software is only designed for certain implant companies and also the availability of the specially designed CAD blocks is considered as a limitation [6, 7]. Lithium disilicate blocks without a screw access channel can be used with other CAD-CAM systems either to restore ti-bases provided by different implant companies or straight preparable abutments [8].

Among the metal free glass-ceramics, lithium disilicate has gained popularity because of its superior esthetics together with favorable mechanical properties [9]. However, the masking ability of the lithium disilicate implant restoration may be compromised by many factors including crown thickness, cement type, and size and material of the abutment used [10, 11]. Many studies investigated the masking ability of lithium disilicate material over metallic substrate and ti-bases. It was concluded that increasing the thickness of the lithium disilicate core reduces the color difference and consequently increases its masking ability [12, 13].

The marginal fit of the crown to the abutment is an important factor when determining the long-term prognosis of an implant-supported fixed dental prostheses [14]. Marginal misfit may increase plaque accumulation along the margins, leading to inflammatory peri-implant disease and subsequent alveolar bone loss. There is no evidence-based consensus regarding a specific clinically tolerable marginal gap, although a marginal fit of between 25 and 40 μm for cemented restorations was considered clinically acceptable [15, 16].

When compared to other ceramics, lithium disilicate material has a high flexural strength, modulus of elasticity, and fracture rate. Multiple studies were conducted to evaluate the fracture resistance of lithium disilicate crowns with screw access channels. They concluded that the presence of an access channel does not reduce the fracture resistance of ceramic crowns [2, 17−19].

The present study aimed to determine whether there is a difference in the masking ability, marginal adaptation, and fracture resistance of lithium disilicate crowns cemented to ti-bases versus straight preparable abutments. The null hypothesis was that no significant difference would be found between the two abutment groups.

## Methods

**Fig. 1:**
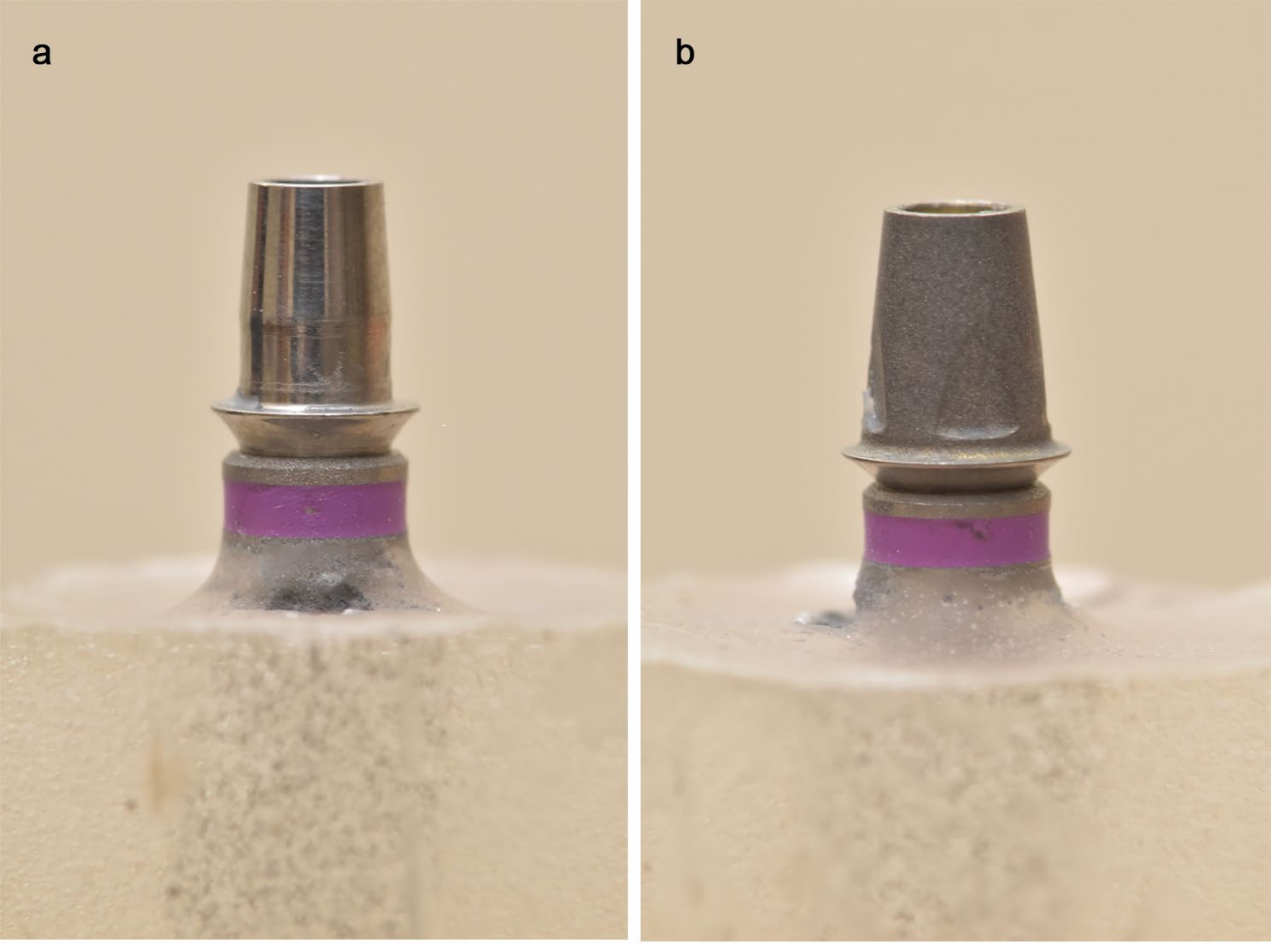
Abutments tightened to implant analogs embedded into resin blocks. **A**, Ti-base. **B**, Airborne abraded straight titanium preparable abutment

Twenty Ø4.1-mm implant laboratory analogs (Straumann Bone Level, Straumann Co, Switzerland) were embedded into Ø2-cm epoxy resin blocks [3] that were randomly divided into 2 groups (n = 10 each) by using a computer-generated list of random numbers (www.randomizer.org). Preparable abutment group: Straight titanium preparable abutments (NNC cementable Abutment, Straumann Co, Switzerland) Ø5 × 5.5 mm. Ti-base group: ti-bases (RN Ti-base; Dentsply Sirona, Straumann Co, Switzerland) Ø4.5 × 4.7-mm (Fig. 1). Preparable abutments were airborne particle abraded by using 50-mm Al_2_O_3_ (Aluminium oxide Eisenbacher Dentalwaren; ED GmbH, Germany).

In the preparable abutment group, abutments were tightened onto the analogs and their screw access channels blocked with a barrier material (Liquidam; Discuss Dental, LLC). In the ti-base group, ti-bases were tightened to the implant analogs and scan bodies (Sirona scanbody; Dentsply Sirona, Switzerland) were snapped over the abutments. A laboratory scanner (InEos X5 lab scanner, Dentsply Sirona) was used to scan all specimens.

**Fig. 2:**
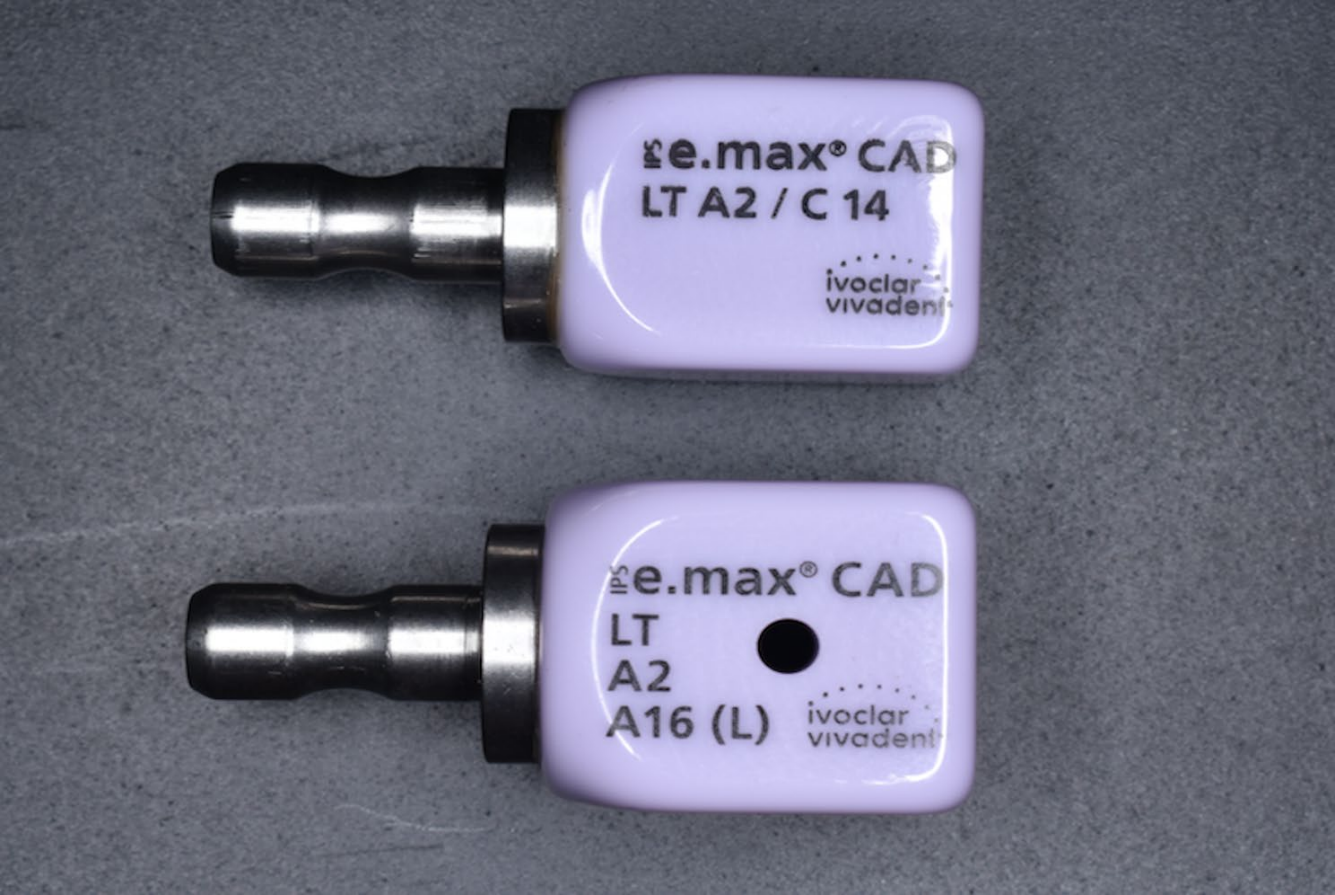
Lithium disilicate CAD blocks used for crown fabrication. **A**, Solid CAD blocks. **B**, CEREC CAD blocks with prefabricated screw access channel

A CAD software program (CEREC software; Dentsply Sirona) was used to design the crowns for both groups standardizing the crown parameters. All crowns were milled (CEREC MC X5; Dentsply Sirona) from lithium disilicate CAD blocks having A2 shade (IPS e.max CAD; Ivoclar AG, Liechtenstein). Specially designed CAD blocks with prefabricated screw access channel were used for the ti-base group (IPS e.max CAD CER/INLAB; Ivoclar AG, Liechtenstein ) while solid CAD blocks were used for the preparable abutment group and screw access channels were prepared later after milling of the crowns and before crystallization (Fig. 2). All crowns have the same design of the mandibular first molar. Same dimensions were used in both groups.

**Fig. 3:**
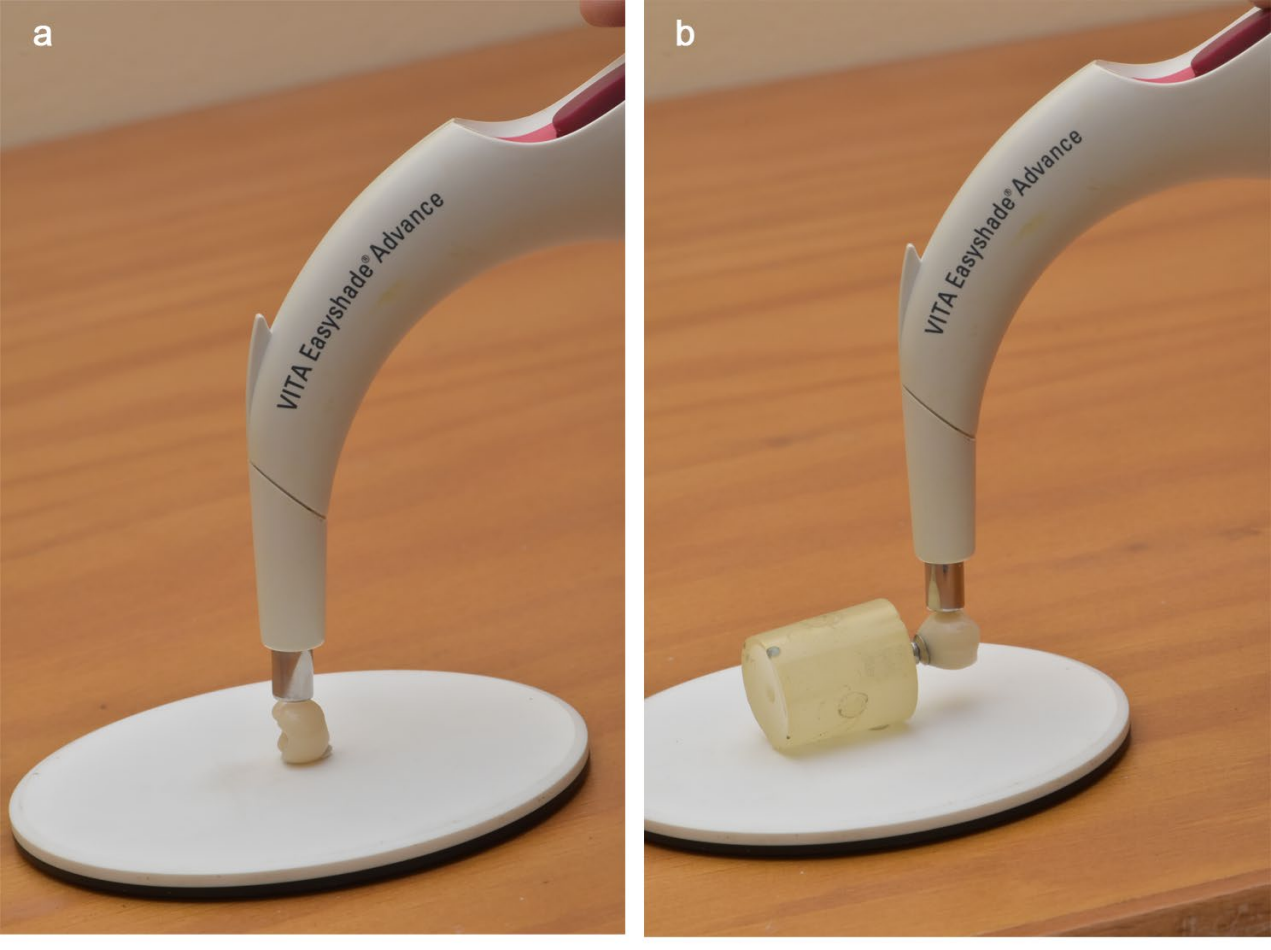
A spectrophotometer was used to measure the shade of the specimens. **A**, Before cementation. **B**, After cementation

Prior to cementation, a spectrophotometer (Easy shade1, Vita, Germany) was used to measure the shade of the crown specimen from 4 different aspects (buccal, lingual, mesial, distal) evaluating the degree of perceptible color based on 3 coordinates; (l*, a* b*) (Fig. 3) [10, 11].

Cementation was done by using a translucent shade dual-polymerizing self-adhesive resin cement (Panavia SA Cement universal; Kuraray Co, Japan) following the manufacturer’s instructions. The technique of crowns cementation to their corresponding abutments mentioned by Khamis MM et al. [2] was followed in the present study. A loading apparatus was used to apply 49-N static load to the crowns during the setting of the resin cement [2].

The spectrophotometer was used again after cementation to measure the shade of the specimens (Fig. 3). To evaluate the masking ability, the color difference of the lithium disilicate crown was calculated before and after cementation using the equation: ΔE*ab = [(L*2 − L*1)2+(a*2 − a*1)2+(b*2 − b*1)2]1/2 [10, 11].

**Fig. 4:**
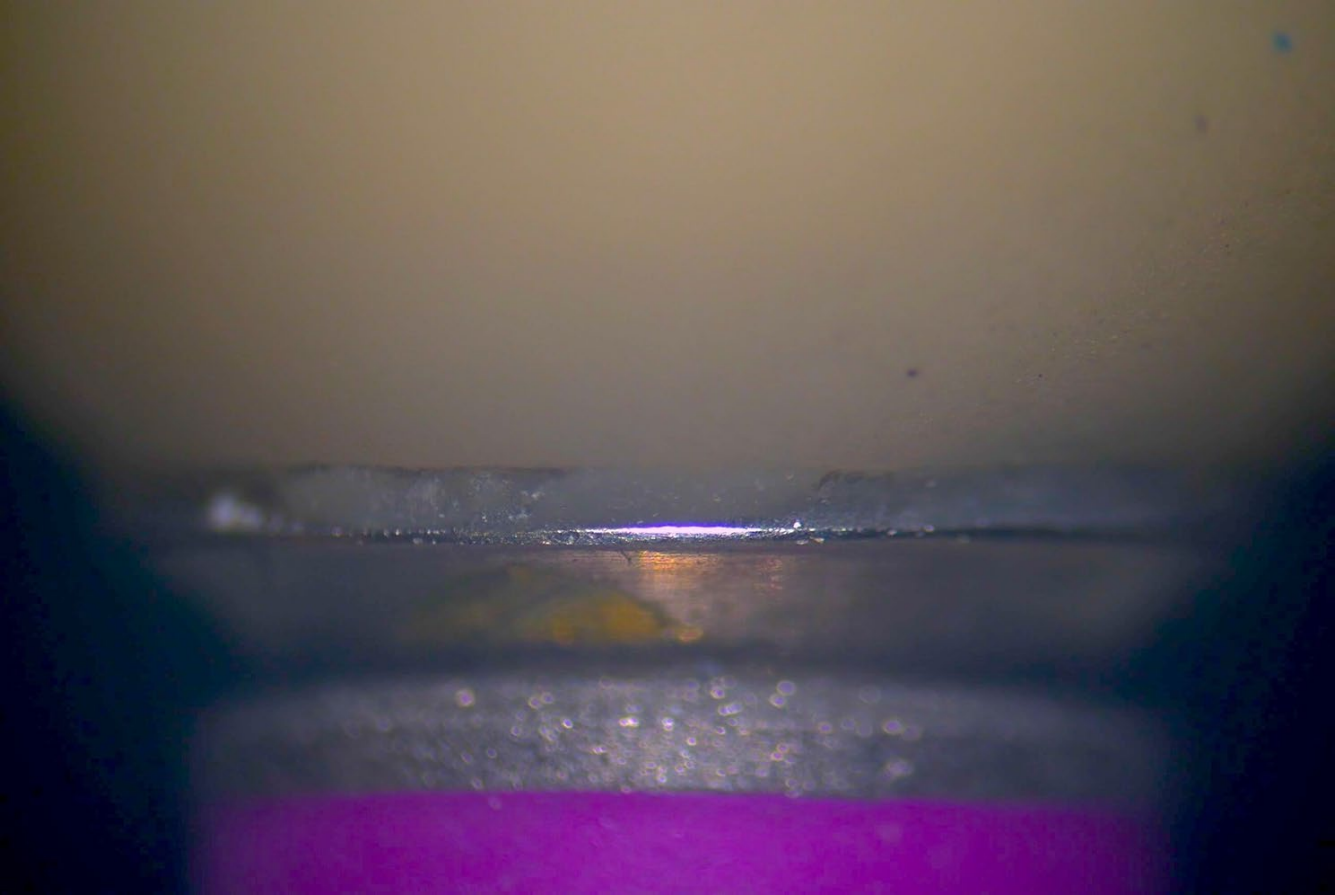
Measurement of the marginal gap by using stereomicroscope at X50 magnification

The vertical distance between the abutment shoulder and the most apical part of the crown represents the marginal gap [15]. The vertical distance was measured before the crown cementation in 4 aspects (Mid-mesial, Mid-distal, Mid-buccal and Mid-lingual) by using stereomicroscope (SZ1145TR; Olympus) at X50 magnification. The stereomicroscope was equipped with a digital camera (ToupCam; ToupTek Photonics, China) and analyzing software (ToupView; ToupTek Photonics, China) (Fig. 4).

The specimens were then aged using a thermocycling machine made specifically for the purpose (Dental biomaterials Department, Alexandria University, Egypt) for 2000 cycles, or 3 months of clinical service, in water baths between 5 and 55 degrees Celsius with dwell times of 1 min in each bath and relaxation periods of 30 s in air between the 2 baths [20]. The specimens were subsequently secured to a specially designed cyclic loading apparatus (Dental biomaterials Department, Alexandria University, Egypt) and loaded with an average functional masticatory force of 50 N over a mean of 120 000 cycles [21].

**Fig. 5:**
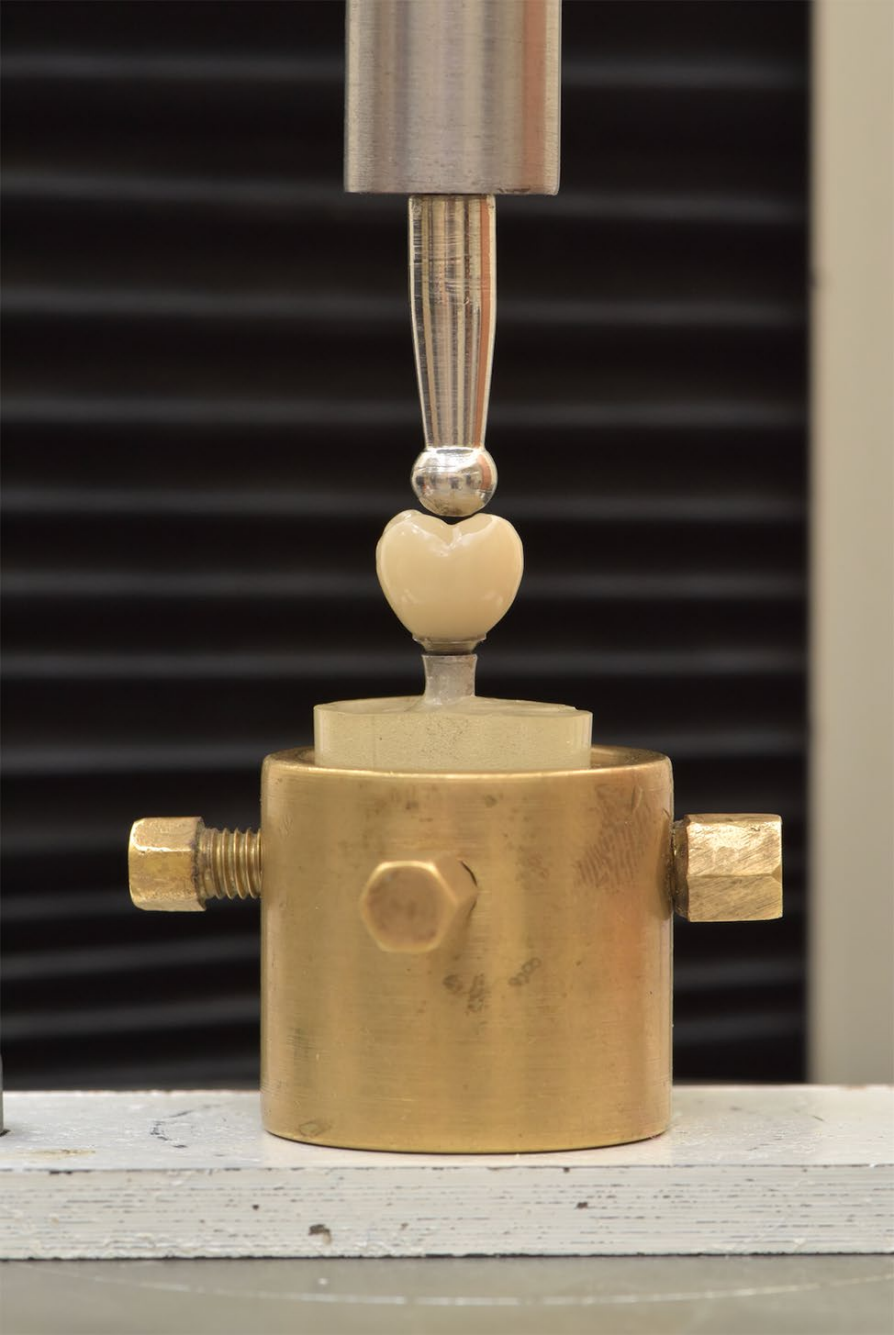
Specimen mounted on a universal testing machine to measure the tensile load required to fracture the crown

After aging, stereomicroscopy was used to evaluate the effect of thermocycling and cyclic loading on the marginal adaptation of the specimens. The samples were then transferred and mounted on a universal testing machine (Department of dental biomaterials, Alexandria University, Egypt) set at 0.5 mm per minute cross head speed and the test for fracture resistance was performed. A holder was constructed with 4 retentive screws to hold the epoxy resin blocks with opposing holes in the epoxy resin blocks to accommodate the retentive screws. A specially designed ball head attachment with dimensions equal to the occlusal diameter of the crowns was fabricated and mounted on the universal testing machine resting on all the cusps and deflecting them when the test starts (Fig. 5). The load required to fracture the restorations was recorded in Newtons (N).

Data were collected and statistically analyzed with a statistical software program (IBM SPSS Statistics for Windows, v23.0; IBM Corp). Normality was checked for all variables by using the Shapiro–Wilk test [22]. Regarding the results of the masking ability and the fracture resistance, all variables showed normal distribution, so means and standard deviations (SD) were calculated, and parametric tests were used. Comparison between the 2 study groups was done by using independent samples t-test. Significance was set at *P* < .05. Regarding the marginal adaptation values, all variables showed non-normal distribution. Means, standard deviations (SD), median and interquartile range (IQR) were calculated. Comparison between the 2 study groups was done using Mann-Whitney U test. Comparison between different timepoints within each study group was done using Friedman test, followed by multiple pairwise comparisons (in case of significant results) by using Bonferroni adjusted significance level P < .05.

## Results

The means and standard deviations of the color difference (∆E) values recorded in the studied groups were presented in Table 1. Comparison between the 2 study groups was done by using independent samples t-test. Significance was set at (*P* < .05). The color difference (∆E) values showed no statistically significant difference between the ti-base group (2.6 ± 0.2) and the preparable abutment group (2.6 ± 0.3) (*P* = .888). The means and standard deviations of the microgap values recorded in the studied groups were presented in Tables 2 and 3. Comparison between the 2 study groups was done using Mann-Whitney U test. Comparison between different timepoints within each study group was done using Friedman test, followed by multiple pairwise comparisons (in case of significant results) using Bonferroni adjusted significance level (*P* < .05).The average of the microgap values was greater in the ti-base group after cementation (13.9 ± 9.2) than the preparable group (7.63 ± 1.78) with no statistically significant difference between the 2 groups (*P* = .49) as presented in Table 2. After cyclic loading and thermocycling, the average of the microgap values (µm) was statistically greater in ti-base group (21.3 ± 7.4) than preparable group (13.3 ± 1.5) (*P* = .02) as presented in Table 3. Regarding the fracture resistance test, the means and standard deviations of the load required to fracture the specimen in (N) recorded in the studied groups were presented in Table 4. Comparison between the 2 study groups was done using independent samples t-test. Significance was set at (*P* < .05). The load required to fracture the specimen in (N) was greater in the preparable group (1671.5 ± 143.9) than in ti-base group (1550.2 ± 157.5) with no statistically significant difference between the 2 groups.

**Table 1:** Mean values and standard deviations of the color that the color difference (∆E) obtained from the 2 groups

	Preparable abutment group	Ti-base group	*P* value
(∆E) Mean ± standard deviation	(2.6 ± 0.3)	(2.6 ± 0.2)	0.888

Significance was set at *P* < .05

**Table 2:** Mean values and standard deviations of the microgap values (µm) obtained from the 2 studied groups after cementation

	Preparable abutment group	Ti-base group	*P* value
Average Marginal gap (µm) Mean ± standard deviation	7.6 ± 1.8	13.9 ± 9.2	0.49

Mann-Whitney test was used

*Statistically significant at *P* < .05

**Table 3:** Mean values and standard deviations of the microgap values (µm) obtained from the two studied groups after thermocycling and cyclic loading

	Preparable abutment group	Ti-base group	*P* value
Average Marginal gap (µm) Mean ± standard deviation	13.3 ± 1.5	21.3 ± 7.4	0.02*

Mann-Whitney test was used

*Statistically significant at *P* < .05

**Table 4:** Mean values and standard deviations of load required to fracture the specimens in the 2 groups

	Preparable abutment group	Ti-base group	*P* value
Load (N) Mean ± standard deviation	1671.5 ± 143.9	1550.2 ± 157.5	0.089

Significance was set at *P* < .05

## Discussion 

The choice of a proper implant abutment for single unit restorations is dependent on many factors. The masking ability, marginal adaptation, and fracture resistance of the restoration are among the factors to be considered. In the present study, the null hypothesis was partially rejected as the marginal gap was only significantly different between the ti-base group and the preparable abutment group after cyclic loading and thermocycling. However, no statistically significant difference was found between the 2 groups regarding the masking ability and the fracture resistance of the restorations. Lithium disilicate restorations were selected in the present study as they have shown favorable esthetic and biomechanical properties in single unit implant replacements in previous studies and ZrO2 can be an alternative [9–11].

The perception of color differences varies among individuals. The literature provides varying values for the perceptible and acceptable color difference thresholds. The perceptible threshold ΔE ranges from 1.0 to 3.7 and the acceptable ΔE threshold ranges from 1.7 to 6.8 to the human teeth and gingiva respectively [23, 24]. In the present study, the perceptible color change was found ΔE < 3.0 in both groups which is considered clinically acceptable and gives no superiority to any of the techniques when esthetics is of concern [10, 11]. The results of the present study was in accordance to Thoma D. et al. [23] who concluded that the median threshold values are 1.8 for the human teeth. The selected preparable abutments in the current study had diameters similar to the ti-bases. The thickness of the crowns for both groups was therefore similar explaining the insignificant differences in shade change. The results of the present study were consistent with the results of other studies assessing the ability of different thicknesses of monolithic lithium disilicate to mask the grey shadow of titanium abutments [10–12].

The results of the present study showed no significant difference regarding the fracture resistance between the crowns cemented to ti-bases in comparison to preparable abutments. The used preparable abutments in the current study had vertical heights similar to the ti-bases. The occlusal thickness of the crowns for both groups was therefore similar explaining the insignificant differences in fracture resistance. Those results were in accordance with multiple studies conducted to evaluate the fracture resistance of lithium disilicate crowns with screw access channels cemented to different types of abutments [2, 7−19].

Marginal fit is one of the most important technical factors for the long-term success of restorations [14]. The results of the present study showed a greater marginal gap in the ti-basegroup when compared to the preparable abutment group. Those results contradict the claim that the combination of ti-bases together with their specially designed lithium disilicate blocks would provide better marginal adaptation than the combination of preparable abutment with regular CAD blocks [4, 5]. However, the average of the marginal gap was considered clinically acceptable in both groups even after thermocycling and cyclic loading [15, 16].

Limitations of the present study include the in vitro designstandardizing all the variables that is not possible in the clinical practice. An in vivo study should be conducted to verify the findings. Also the small sample size. Further studies should be conducted with larger sample size with more types of the cement used and different heights of the preparable abutments.

## Conclusion

Within the limitations of the current in-vitro study … Based on the findings of this in vitro study, the following conclusions were drawn:

1. The 2 types of abutments used in the present study did not compromise the masking ability of the screw-retained lithium disilicate implant-supported crowns cemented to them.
2. The marginal accuracy achieved for the 2 groups was within the range of clinical acceptance.
3. Crowns fabricated over ati-base together with their special lithium disilicate block with a prefabricated screw access channel did not ensure greater fracture resistance or better marginal adaptation when compared with preparable abutments.

## Data Availability

The raw data of the present study is available at:https://figshare.com/articles/dataset/Fracture_Resistance_Masking_Ability_and_Marginak_Adaptation/23269352.

## Abbreviations

Ti-base: Titanium bases
N: Newtons
CAD-CAM: computer-aided design and computer-aided manufacturing
